# Systematic Bioinformatic Approach for Prediction of Linear B-Cell Epitopes on Dengue E and prM Protein

**DOI:** 10.1155/2016/1373157

**Published:** 2016-09-01

**Authors:** Mahesha N. Nadugala, Prasad H. Premaratne, Charitha L. Goonasekara

**Affiliations:** Faculty of Medicine, General Sir John Kotelawala Defence University, 10390 Ratmalana, Sri Lanka

## Abstract

B-cell epitopes on the envelope (E) and premembrane (prM) proteins of dengue virus (DENV) were predicted using bioinformatics tools, BepiPred, Ellipro, and SVMTriP. Predicted epitopes, 32 and 17 for E and prM proteins, respectively, were then characterized for their level of conservations. The epitopes, EP4/E (48–55), epitope number 4 of E protein at amino acids 48–55, EP9/E (165–182), EP11/E (218–233), EP20/E (322–349), EP21/E (326–353), EP23/E (356–365), and EP25/E (380–386), showed a high intraserotype conservancy with very low pan-serotype conservancy, demonstrating a potential target as serotype specific diagnostic markers. EP3 (30–41) located in domain-I and EP26/E (393–409), EP27/E (416–435), EP28/E (417–430) located in the stem region of E protein, and EP8/prM (93–112) from the prM protein have a pan-serotype conservancy higher than 70%. These epitopes indicate a potential use as universal vaccine candidates, subjected to verification of their potential in viral neutralization. EP2/E (16–21), EP5/E (62–123), EP6/E (63–89), EP19/E (310–329), and EP24/E (371–402), which have more than 50% pan-serotype conservancies, were found on E protein regions that are important in host cell attachment. Previous studies further show evidence for some of these epitopes to generate cross-reactive neutralizing antibodies, indicating their importance in antiviral strategies for DENV. This study suggests that bioinformatic approaches are attractive first line of screening for identification of linear B-cell epitopes.

## 1. Introduction

Dengue is a mosquito-borne systemic viral infection caused by any of the four antigenically related dengue viruses (DENV). An estimated 400 million people worldwide are infected with dengue annually, leading to approximately 100 million cases of dengue and 21,000 deaths [1]. People infected with DENV can be asymptomatic or develop symptoms that range from a mild fever to severe Dengue Hemorrhagic Fever (DHF) and Dengue Shock Syndrome (DSS). A dengue-naïve individual exposed to a primary infection develops long-lasting protective immunity only to the infecting serotype [2]. A second infection with a new serotype increases the risk of developing DHF/DSS. The presence of cross-reactive but weakly neutralizing antibodies (NAbs) induced following the primary infection has been hypothesized to be a cause for DHF or DSS through a mechanism known as Antibody Dependent Enhancement (ADE) [3].

DENV is a positive-sense, single-stranded RNA virus containing a genome of approximately 10.6 kb. The single open reading frame encodes a polyprotein precursor, which is cleaved by cellular and viral proteases into three structural proteins, capsid (C), precursor membrane (prM), and envelope (E), and seven nonstructural proteins [4]. The E protein participates in cell recognition and cell entry and is physically arranged in a herringbone pattern as a series of 90 homodimers on the outer surface of the mature virus particle [5]. The E protein consists of three structural domains (D), namely, DI, DII, and DIII [6, 7]. At one end of the molecule is the fusion loop within DII and at the other end is DIII, which is involved in host cell binding [8]. The prM protein has been shown to serve as a chaperon of E protein [9, 10] and to prevent E protein from premature fusion within acidic compartments along the secretary pathway [11, 12]. On immature particles, the prM protein lies over the E protein and serves to protect the virus particle from undergoing premature fusion or inactivation within the secretary pathway of the host cell. The prM is subsequently cleaved by a host protease to release the ectodomain and allow viral maturation [13].

As shown in previous studies, B-cell responses are known to be directed against the viral structural proteins E and prM of DENV [14–19], which are fundamental in the pathogenesis of virus infection. B-cell epitopes of those proteins are therefore targets in the development of effective therapeutic and diagnostic tools [20]. The present study is an initiative of the process of investigating such epitopes from DENV E and prM proteins.

Traditional epitope selection methods are usually cumbersome and require large resources. However, the advent of technologies related to immune epitope prediction and databases could aid the prediction of B-cell epitopes. Sophisticated bioinformatic tools enable the systematic scanning for candidate epitopes from large sets of protein antigens. This approach saves considerable time and cost, especially for researchers in countries with limited resources [21]. In this backdrop, three bioinformatic tools, namely, BepiPred, Ellipro, and SVMTriP, were selected for identifying potential B-cell epitopes of DENV E and prM proteins, for the present study. Further, we focused on prediction of linear B-cell epitopes, as they are more applicable in the development of peptide based vaccines and diagnostic tools [22].

As predicted and analyzed in this study, seven epitopes on the E protein demonstrated the potentiality to be used as serotype specific diagnostic markers. Several epitopes on the E protein and prM proteins were having high dengue group conservancies and located in positions with previous evidence for generating NAbs and therefore indicate a potential use of them in antiviral strategies or in developing as dengue group diagnostic markers.

## 2. Materials and Methods

### 2.1. Retrieving the Protein Sequences

The E and prM protein sequences from 200 variants belonging to all 4 serotypes of DENV (DENV1, DENV2, DENV3, and DENV4) were retrieved from National Center for Biotechnology Information (NCBI) (http://www.ncbi.nlm.nih.gov/). Each serotype consisted of fifty sequences each for both E and prM protein. The retrieved data set is representative of a wide geographical coverage (countries from South Asia, East Asia, America, and Africa, where dengue is prevalent) and a time span of approximately 50 years (isolates from 1963 to 2014). Isolates with partial sequences in NCBI were excluded. The variable and conserved regions were compared among the downloaded isolates after aligning the isolates using Clustal W on MEGA6 (http://www.megasoftware.net/).

### 2.2. Selection of Prediction Tools

B-cell epitope prediction was carried out by use of tools available online. Three tools, BepiPred, Ellipro, and SVMTriP, were selected after a thorough screening of all the currently available free computational tools. These tools were primarily selected on their free accessibility and on epitope prediction characteristics used in the prediction tool.

BepiPred [23] (http://tools.iedb.org/bcell/) is a combination method, produced by combining the predictions of a Hidden Markova model and the propensity scale by Parker et al. [24]. This method assigns a score value to each protein residue. Threshold was set at −0.2 (to obtain the sensitivity of 75% and specificity of 50%, similar to those of SVMTriP) or at 0.35 (the default). The second tool selected, Ellipro [25] (derived from Ellipsoid and Protrusion) (http://tools.iedb.org/ellipro/), is a web-tool that implements a modified version of Thornton's method (regions with high protrusion index values corresponding to continuous epitopes) [26], and together with a residue clustering algorithm, the MODELLER program [27], and the Jmol viewer, it allows the prediction and visualization of antibody epitopes in protein sequences and structures. The following values were selected for Ellipro parameters for prediction of epitopes: blast expectation value: 1, maximum number of 3D structural templates (s): 5, maximum distance (angstrom): 6, and minimum score (cut-off for the selection of epitopes): 0.5. The third tool selected, SVMTriP (http://sysbio.unl.edu/SVMTriP/), utilizes Support Vector Machine in combination with tripeptide similarity and propensity scores (SVMTriP) [28]. The length of predicted epitopes was retained to 20 a.a., to obtain a maximum performance at sensitivity and specificity values of 80% and of 55%, respectively. The lowest score of the recommended epitopes by the tool, which was 0.8, was considered as the cut-off for the selection of epitopes. In summary the three tools described above employ different models like Hidden Markova model and Support Vector Machine model, among others, and consider different amino acid propensities such as hydrophilicity, flexibility, and secondary structure for prediction of B-cell epitopes. Therefore, simultaneous applications of three bioinformatics tools will enable a comprehensive prediction of B-cell epitopes.

### 2.3. Prediction of B-Cell Epitopes

**Figure 1:**
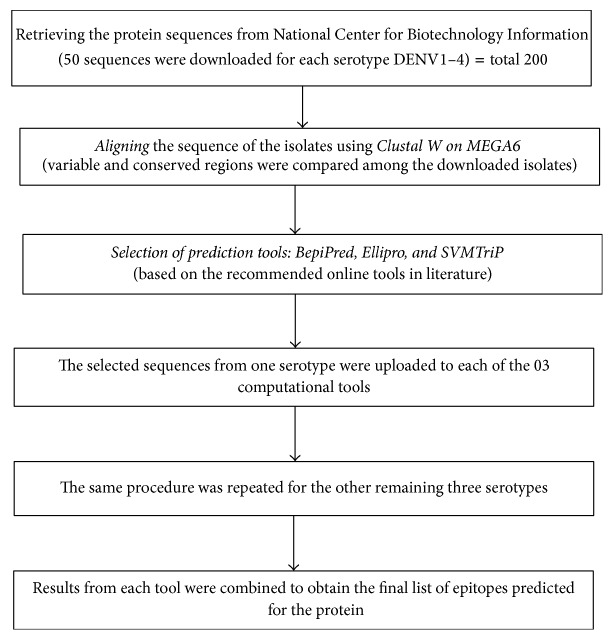
Flow diagram for the method of epitopes prediction.* The same procedure was followed for both proteins (E and prM) separately*.

The selected sequences of the E and prM protein from one serotype were uploaded to each of the 03 computational tools (Figure 1). Results from each tool were combined to obtain the final list of epitopes predicted for the protein. The same procedure was reiterated for the other three serotypes.

### 2.4. Prediction of Epitope Conservancy

**Figure 2:**
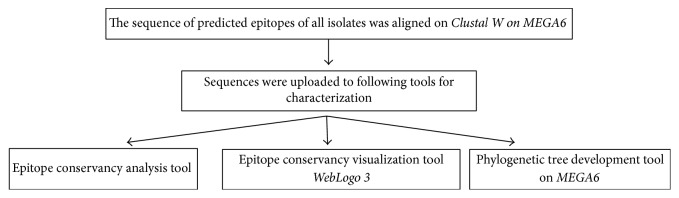
Flow diagram of methodology for epitope analysis.

Conservancy patterns of the entire protein sequence of E and prM proteins and predicted epitopes were determined by the Epitope Conservancy Analysis tool [29] developed by Immune Epitope Database and Analysis Resource (IEDB) (http://www.iedb.org/). Epitopes conservancy was measured at two levels: first within each serotype (intraserotype conservancy) and then among all four serotypes (pan-serotype conservancy) (Figure 2).

### 2.5. Visualization of Conservation

The level of pan-serotype conservancy was visualized using WebLogo 3.0 [30] (http://weblogo.berkeley.edu/logo.cgi) (Figure 2).

### 2.6. Construction of Phylogenetic Tree

The neighbor-joining method on MEGA6 was used to construct phylogenetic tree for each predicted epitope using the multiple sequence alignments (MSA) generated for each epitope (Figure 2).

## 3. Results and Discussion

The epitopes were predicted independently using three selected prediction tools and the results were compared. The predicted epitopes were characterized in terms of their predictability, conservation, phylogenetics, and so forth. Several observations on the significance and the potential use of the predicted epitopes made in the present study are described below.

### 3.1. General Characterization of the Predicted Epitopes

Total of forty-five epitopes of E and prM proteins were predicted by the three tools used. The same protein regions were predicted as epitopes with respect to each serotype with one to two amino acid differences to the length of the epitopes between the serotypes. Thirty-two out of forty-five predicted epitopes are on E protein: seventeen were predicted by BepiPred, eleven by Ellipro, and four by SVMTriP (Table 1). The remaining thirteen epitopes were predicted on prM: five epitopes by BepiPred, six by Ellipro, and two by SVMTriP (Table 2). In order to apply consistent criteria of sensitivity and specificity across the three tools, for BepiPred tool, predictions were carried out at −0.2 thresholds, at which the sensitivity and specificity percentages (75% and 50%, resp.) were the closest to those of SVMTriP with 20 a.a. epitope length (80% and 55%, resp.). When the predictions were also carried out at 0.35-threshold value, with sensitivity and specificity of 49% and 75%, respectively, fewer numbers of epitopes were predicted (8 epitopes for E protein and 4 epitopes for prM). Further, the epitope regions predicted at 0.35 thresholds were also predicted at −0.2 thresholds, at which the overall predictions showed a better agreement with epitopes predicted by other two tools. Therefore BepiPred results shown above are for predictions carried out at the threshold value −0.2.

With most protein locations, although the epitopes predicted are not identical, similar locations have been predicted by the three different tools, as epitopes, such that they are overlapping. The sequences of overlapping epitopes as predicted by the three tools are indicated in Table 3 (for E protein) and Table 4 (for prM protein). Some regions have further been predicted by all the three tools (Tables 3 and 4). For example, EP19/E, EP20/E, and EP21/E; EP27/E, EP28/E, and EP29/E; EP31/E, EP32/E, and EP30/E; EP11/prM, EP10/prM, and EP12/prM are overlapping epitopes predicted by all the three tools, SVMTriP, BepiPred, and Ellipro, respectively. The results, therefore, show a good agreement in the predictions among the three tools. We further noted that some of these predicted epitopes, partially or as full sequence, have been previously shown to generate antibodies. To elaborate on this point, the epitopes EP19/E, EP24/E, EP3/prM, EP6/prM, and EP7/prM partially constitute regions that have been shown to induce natural antibodies [31–34]. These evidences strengthen the potential of bioinformatical tools to predict epitopes, which are antigenic under natural conditions.

The percentage conservancy analysis was carried out on the entire sequence of proteins studied and for the predicted epitopes by IEDB tools. The pan-serotype conservancy ranged from 63% to 100% for both E and prM proteins. But intrastereotypic conservancies of the four serotypes for both proteins resulted in percentages more than 85%. These results indicate the similarity of isolates within a serotype and also give evidences for genetic variability among serotypes.

All the E epitopes (13 in number) showed a pan-serotype conservancy ranging from 10 to 83%, nineteen with pan-serotype conservancy above 50% (Table 1). For prM protein, the pan-serotype conservancy is more than 50% in most of the epitopes (except in EP3/prM, EP5/prM, EP7/prM, EP12/prM, and EP13/prM) but less than 70% (except in EP8/prM). This medium level conservation of prM epitopes observed between the DENV serotypes, could be resulting a cross-reactive antibody binding with each other, which might not be neutralizing. This notation is also suggestive in the recent findings demonstrating anti-prM antibodies of one serotype being highly cross-reactive, without the neutralizing potential against the other serotypes [14], a phenomenon that could lead to ADE.

Several of the predicted epitopes were found located on the surface of the respective protein, with significant functional roles in either host attachment or infection. Some of them were also found highly conserved across the serotypes. Those epitopes with high pan-serotype conservancy and also located on the protein where it is crucial for viral infection would be very interesting. The rationale behind this approach is that conserved epitopes constitute the regions in DENV proteins with minimal or no amino acid differences among different DENV serotypes/variants and therefore are expected to cause the least if not no variability in immune response against different DENV viral serotypes/strains. Thus, such epitopes with neutralizing immunogenicity will be excellent candidates for broadly reactive vaccine development. The neutralization ability of those epitopes, however, needs verification through biochemical investigations, as any significant variability in the immune responses across serotypes, which could be caused by even a single amino acid difference, may lead to the development of ADE rather than protection. On the other hand some of the predicted epitopes were noted to have a low pan-serotype conservancy level, at the same time having high intraserotype conservancies. Such epitopes could be potential candidates for serotype specific diagnostic markers. The characteristics of the epitopes which we have identified as potentially significant in dengue diagnosis and therapeutics are discussed in the following in detail.

### 3.2. Epitopes with Low Pan-Serotype Conservancy but with High Intraserotype Conservancy

**Figure 3:**
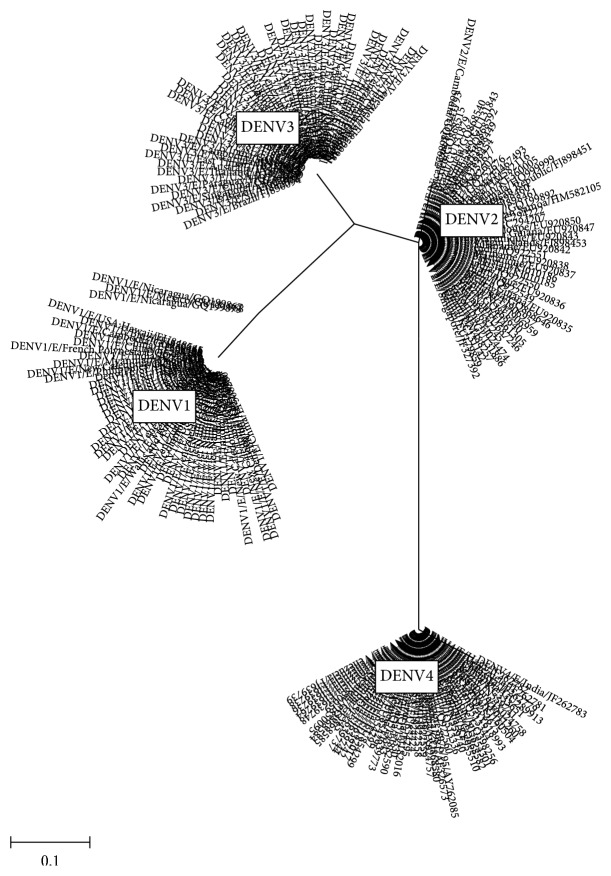
Phylogenetic tree of epitopes EP4/E.

Seven of the thirteen E epitopes with less than 50% pan-serotype conservation level, the epitopes EP4/E, EP9/E, EP11/E, EP20/E, EP21/E, EP23/E, and EP30/E, are with very low pan-serotype conservancy (less than 40%) but high intraserotype conservancies (more than 80%). In particular, the epitopes EP4/E and EP23/E are suggestive to be promising candidates to be serotypic diagnostic marker, owing to their pan-serotype conservancy below 15%. To add on to the evidence, EP4/E and EP9/E of the above are located on DI of E protein. This is complementary with the findings of Roehrig et al. [35] that DI contains predominantly type-specific nonneutralizing epitopes. In addition, phylogenetic analysis of these epitopes (that of EP4/E as shown in Figure 3) showed a clustering pattern with highly isolated and distant clusters for each serotype compared to the serotype clustering pattern for the whole envelop protein sequence. This gives a good evidence for high intraserotype and low pan-serotype conservancy of these epitopes. Yet, the potentialities of all these epitopes will need verification through laboratory tests in order to confirm their use as a serotypic diagnostic marker.

Unlike the above mentioned E protein epitopes, except for EP13/prM (which has a pan-serotype conservancy of 33% and intraserotype conservancy more than 80%), none of the other predicted prM protein epitopes had striking characteristics of a potential candidate for a serotypic diagnostic marker, as determined through conservation analysis in this study. The epitope EP7/prM (Ellipro), corresponding to the peptide region 55–65 a.a. on the prM protein, showed a pan-serotype conservancy of 18%. However it only showed higher intraserotype conservancies for DENV2 (81%), DENV3 (81%), and DENV4 (90%), whereas it was only 36% for DENV1. Therefore these epitopes could be useful in the differentiation of the former serotypes. A previous study on DENV infected mice and human using prM protein of DENV2 has established the production of specific antibodies against the region 57-71 a.a., indicating the potential use of EP7/prM in specific identification of certain serotypes [34]. This evidence further reinforces the potentiality of the computer based predictions of protein epitopes. EP3/prM representing the amino acid sequence from 15 to 22 a.a. on the prM protein also showed a lower pan-serotype conservancy of 25% and higher intraserotype conservancy of 87% for three serotypes, DENV1, DENV3, and DENV4. In DENV2, the intraserotype conservancy for this epitope is only 37%. A previous study has concluded that the peptide sequences from 19 to 34 a.a. of prM of DENV2 protein, which partially overlaps with EP3/prM, elicit high titer antibodies in Balb/c mice. This epitope also reacts with sera from DENV2 infected patients, suggesting that specific antibodies against the epitope were elicited in both DENV infected mice and human [33]. However, the same study observes a broad cross-reactivity and poor neutralizing activity but potent ADE activity in this epitope toward the four DENV serotypes and immature DENV. Luo et al. also find 14–8 a.a. region of prM protein, as an infection enhancing epitope [36]. Better understanding of EP3/prM could provide new insight into the pathogenesis of DENV infection.

### 3.3. Epitopes of Highly Conserved Regions

EP5/E (62–123 a.a.) predicted by Ellipro includes highly conserved fusion loop (FL) (97–111 a.a.) and bc loop (73–79 a.a.). EP6/E (63–89 a.a.) which is predicted by BepiPred contains bc loop within the peptide stretch. Further EP7/E (97–108) predicted by the same tool is more or less within the fusion loop. The above epitopes, which are located on the DII of E protein, show pan-serotype conservancies of 64%, 67%, and 83%, respectively. According to Rey et al. [6] and Roehrig et al. [35], DII contains many cross-reactive epitopes eliciting neutralizing and nonneutralizing monoclonal antibodies to fusion peptides. The most significant fusion loop amino acid residues that reduce the binding of human monoclonal antibodies (hMABs) to E protein are W101, L-107, and/or G109 [37]. EP5/E contains these three amino acid residues. hMABs directed against the highly conserved fusion loop block viral entry by inhibiting E protein mediated fusion [37]. The antibodies that recognize bc loop have several desirable features, neutralize DENV effectively, and compete for binding against more common low-potency FL antibodies, believed to contribute to antibody-mediated disease [38]. Hence, characterization of EP5/E that contains both fusion and bc loop regions, EP6/E that contains bc loop, and EP7/E that contains the fusion loop may provide new insights into DENV vaccines and therapeutic strategies.

Seven epitopes have been identified in the C-terminus of E protein, where there are two *α* helices (EH1 and EH2) in the stem region (396–452 a.a.) and two transmembrane domains (ET1 and ET2) in the anchor region (452–495 a.a.) [38]. Epitopes at the C-terminus are positioned at 399–405 a.a. (EP26/E), 416–435 a.a. (EP27/E), 417–430 a.a. (EP28/E), 425–445 a.a. (EP29/E), 454–488 a.a. (EP30/E), 459–478 a.a. (EP31/E), and 469–475 a.a. (EP32/E). The epitope sequence 416–435 a.a. (EP27/E) showed a pan-serotype conservancy of 75% and 95% of intraserotype conservancy. EH1 and EH2 domains are involved in both assembly and entry steps of the DENV replication cycle; this feature, together with the high degree of sequence conservation, suggests that the stem region represented by EP27/E is a potential target of a universal vaccine candidate, if it also induces the production of neutralizing antibodies.

Analysis of WebLogo revealed a partially conserved region on prM protein spanning from 65 to 117 a.a., which includes EP8/prM and EP9/prM. It is worth noting that EP9/prM showed a pan-serotype conservancy of 75% and an intraserotype conservancy higher than 90% within each of the four serotypes. This result suggests that this epitope could be a potential universal vaccine candidate, if it also proves to be neutralizing upon verification with laboratory experiments.

### 3.4. Other Predicted Epitopes with Significance

Several other epitopes are located on the protein at locations suggestive to be important in viral infection, host cell binding, and so forth. As such, DIII of E protein is the putative receptor binding domain based on several factors: DII has an immunoglobulin-like fold characteristic of many cell receptors, DIII has loops that project further from the virion surface than either DI or DII, and various soluble forms of DIII have been shown to block infection of cells by DENV [18]. Peptide sequence spanning from 309 to 320 has been recognized as a highly conserved linear epitope on AB loop of the DIII [31]. The same region has also been predicted in our study as a part of EP19/E, which showed a pan-serotype conservancy of 55% and intraserotype conservancy of 80%. However three-dimensional modeling analysis done by Li et al. [31] suggests that this epitope is surface exposed on DIII but less accessible on the surface of the E protein dimer and trimer, especially on the surface of the mature virion, therefore being poorly neutralizing. Further characterization of this epitope using laboratory tests would validate the above suggestion.

Some of the predicted epitopes are located partially on two different domain regions: EP17/E (287–313 a.a.) is located partially on DI and partially on DIII and EP24/E (371–402 a.a.) is partially on DIII and stem region. EP26/E (393–409 a.a.) represents the DENV complex conserved peptide 393-KKGSSIGQ/KM-401 [32]. The sequence 393–401 is implicated in cell binding and sequence 401–413 is implicated as involved in E protein homotrimer formation [39]. This may make this sequence alone, or possibly as a discontinuous epitope with the adjacent 304–313 sequences, useful for diagnostic assays as well as for generating active cross-protection against all serotypes of dengue [40].

Finally, we have visualized all the epitope sequences of E and prM proteins on WebLogo, as shown in Tables 5 and 6, which gave clear understanding of amino acid composition at each position of the epitopes with reference to isolate used for the study. This would mainly help in deciding the most appropriate generalized sequence for epitope synthesis for laboratory assays, as the next step of validating the important epitopes, which were predicted in the current study.

## 4. Conclusion

This study concludes that the bioinformatic approach is an effective initial step to screen potential linear epitopes of DENV E and prM proteins. These predicted epitopes, however, need verification through experimental approaches in order to confirm their immunogenicity and neutralization abilities, before confirming their potential use in diagnostic or therapeutic applications. According to the analysis of the current study, the epitopes, predicted bioinformatically, prove promising being carried to the next step of experimental verification as future work.

## Figures and Tables

**Table 1:** Epitopes predicted by bioinformatics on E protein with conservancy results.

ID of the predicted epitope	^#^Epitope sequence	Epitope length (a.a.)	Method of prediction (score)	Percentage conservancy (min%)				
				Intraserotype				Pan-serotype
				DENV1	DENV2	DENV3	DENV4	
*Entire E protein sequence*				96	93	97	95	63
EP1/E	SRDFVEGLSGATW ^*∗*^8–20/DI	13	BepiPred (−1.6–0.26)	84	100	100	92	69
EP2/E	SGATWV ^*∗*^16–21/DI	6	Ellipro (0.5)	83	100	100	83	66
EP3/E	CVTTMAKDKPTL ^*∗*^30–41/DI	12	BepiPred (−0.06–0.95)	75	92	92	100	75
EP4/E	TEVTNPAV ^*∗*^48–55/DI&DII	8	BepiPred (−0.13–0.71)	88	88	100	88	13
EP5/E	EAKISNTTTDSRCPTQGEATLVEEQDANFV CRRTFVDRGWGNGCGLFGKGSLITCAKFKCVT ^*∗*^62–123/DII	62	Ellipro (0.8)	96	93	96	69	64
EP6/E	AKISNTTTDSRCPTQGEATLVEEQDAN ^*∗*^63–89/DII	27	BepiPred (−0.12–1.6)	96	93	93	93	67
EP7/E	VDRGWGNGCGLF ^*∗*^97–108/DII	12	BepiPred (−0.05–0.87)	100	83	100	100	83
EP8/E	HTGDQHQVGNESTEHGTTATITPQAPTTEIQLT ^*∗*^144–176/DI	33	BepiPred (0.12–1.7)	88	85	88	90	42
EP9/E	PQAPTTEIQLTDYGALTL ^*∗*^165–182/DI	17	Ellipro (0.6)	88	94	88	94	35
EP10/E	ALTLDCSPRTGLD ^*∗*^180–192/DI	13	BepiPred (−0.05–0.9)	100	85	100	100	54
Ep11/E	LPWTSGASTSQETWNR ^*∗*^218–233/DII	16	BepiPred (0.06–1.8)	94	88	88	81	37
EP12/E	GASTSQETW ^*∗*^223–231/DII	9	Ellipro (0.6)	88	77	77	77	44
EP13/E	LVTFKTAHAKKQEVVVLGS ^*∗*^237–255/DII	19	Ellipro (0.7)	94	94	100	84	63
EP14/E	TAHAKKQ ^*∗*^242–248/DII	7	BepiPred (−0.04–0.78)	86	86	100	100	43
EP15/E	VLGSQEGAMHTALTGATEIQTSGTTTI ^*∗*^252–278/DII & DI	27	BepiPred (−0.03–1.66)	93	96	96	89	55
EP16/E	FAGHLKCRLKMDKLTLKGMS ^*∗*^279–298/DI	20	SVMTriP20 (0.9)	90	100	85	95	65
EP17/E	LKMDKLTLKGMSYVMCTGSFKLEKEVA ^*∗*^287–313/DI&DIII	27	Ellipro (0.6)	92	88	92	96	48
EP18/E	FKLEKEVAETQHGT ^*∗*^306–319/DIII	14	BepiPred (−0.14–0.95)	100	71	100	93	50
^¥^EP19/E	KEVAETQHGTVLVQIKYEGT ^*∗*^310–329/DIII	20	SVMTriP20 (1.0)	90	80	95	85	55
EP20/E	VQIKYEGTDAPCKIPFSTQDEKGVTQNG ^*∗*^322–349/DIII	28	Ellipro (0.7)	92	82	96	85	35
EP21/E	YEGTDAPCKIPFSTQDEKGVTQNGRLIT ^*∗*^326–353/DII	28	BepiPred (−0.16–1.5)	93	86	96	82	36
EP22/E	PIVTDKEKPVNIEAEPPFGES ^*∗*^356–376/DIII	21	BepiPred (−0.12–1.7)	86	86	90	90	43
EP23/E	PIVTDKEKPV ^*∗*^356–365/DIII	10	Ellipro (0.6)	80	80	80	80	10
^*¥*^EP24/E	PPFGESYIVIGAGEKALKLSWFKKGSSIGKMF ^*∗*^371–402/DIII & Stem	32	Ellipro (0.6)	93	81	93	93	59
EP25/E	IGAGEKA ^*∗*^380–386/DIII	7	BepiPred (−0.03–0.2)	71	86	71	71	29
EP26/E	KKGSSIGKMFEATARGA ^*∗*^393–409/C-terminus	17	BepiPred (−0.04–0.6)	94	88	94	88	71
EP27/E	GDTAWDFGSIGGVFTSVGKL ^*∗*^416–435/C-terminus	20	SVMTriP20 (0.9)	95	95	95	95	75
EP28/E	DTAWDFGSIGGVFT ^*∗*^417–430/C-terminus	14	BepiPred (−0.06–0.6)	86	92	100	92	71
EP29/E	IGGVFTSVGKLVHQIFGTAYG ^*∗*^425–445/C-terminus	21	Ellipro (0.6)	85	95	91	95	55
EP30/E	TMKIGIGVLLTWLGLNSRSTSLSMTCIAVGLITLY ^*∗*^454–488/C-terminus	35	Ellipro (0.8)	91	91	94	88	42
EP31/E	IGVLLTWLGLNSRSTSLSM ^*∗*^459–478/C-terminus	19	SVMTriP20 (0.8)	89	94	94	94	52
EP32/E	NSRSTSL ^*∗*^469–475/C-terminus	7	BepiPred (−0.14–0.2)	86	100	86	100	57

^*∗*^Sequence position/domain. ^#^Epitope sequence is given with reference to DENV1 isolate AY713476. ^*¥*^Epitopes that have been previously described by other authors (cited in the Discussion).

**Table 2:** Epitopes predicted by bioinformatics on prM protein with conservancy results.

ID of the predicted epitope	^#^Epitope sequence	Epitope length (a.a.)	Method of prediction (score)	Percentage conservancy (min%)				
				Intraserotype				Pan-serotype
				DENV1	DENV2	DENV3	DENV4	
*Entire prM protein sequence*				93	87	98	96	63
EP1/prM	FHLTTRGGE ^*∗*^1–9	9	Ellipro (0.6)	88	77	88	88	55
^*¥*^EP2/prM	TTRGGEPHMIVSKQERG ^*∗*^4–20	17	BepiPred (0.05–1.1)	88	71	94	94	59
^*¥*^EP3/prM	SKQERGKS ^*∗*^15–22	8	Ellipro (0.5)	87	37	87	87	25
EP4/prM	KTAEG ^*∗*^26–30	5	Ellipro (0.5)	80	60	100	100	60
EP5/prM	IAMDL ^*∗*^37–41	5	Ellipro (0.5)	100	40	100	80	40
EP6/prM	LCEDTMTYKCPRITEAEPDDVDCWCNATDTWVTYGTCSQTGEHRRDKRSV ^*∗*^44–93	50	BepiPred (−0.02–1.7)	82	92	96	96	68
^*¥*^EP7/prM	RITEAEPDDVD ^*∗*^55–65	11	Ellipro (0.6)	36	81	81	90	18
EP8/prM	VALAPHVGLGLETRTETWMS ^*∗*^93–112	20	SVMTriP20 (1.0)	95	100	95	90	75
EP9/prM	LETRTETWMSSEGAWKQIQKV ^*∗*^103–123	21	BepiPred (−0.04–0.8)	90	95	95	95	57
EP10/prM	TWALR ^*∗*^125–129	5	BepiPred (−0.09–0.1)	80	80	80	100	60
EP11/prM	ALRHPGFTIALFLAHAIGT ^*∗*^127–146	20	SVMTriP20 (0.8)	90	95	85	90	50
EP12/prM	GAWKQIQRVETWALRHPGFTVILALFLAH AIGTSITQKGIIFILLMLVTPS ^*∗*^115–165	50	Ellipro (0.7)	86	80	94	94	46
EP13/prM	GTSITQ ^*∗*^145–150	6	BepiPred (−0.06–0.4)	100	83	83	100	33

^*∗*^Sequence position. ^#^Epitope sequence is given with reference to DENV1 isolate AY713476. ^*¥*^Epitopes that have been previously described by other authors (cited in the Discussion).

**Table 3:** Overlapping epitopes on the E protein.

BepiPred	Ellipro	SVMTriP
^8^SRDFVEGL***SGATW*** ^**20**^ EP1/E	^16^ ***SGATW***V^21^ EP2/E	NP

^**63**^ ***AKISNTTTDSRCPTQGEATLVEEQDAN*** ^**89**^ EP6/E	^**62**^E***AKISNTTTDSRCPTQGEATLVEEQDAN***FVCRRTF***VDRGWGNGCGLF***GKGSLITCAKFKCVT^123^ EP5/E	NP
^**97**^ ***VDRGWGNGCGLF*** ^**108**^ EP7/E		

^144^HTGDQHQVGNESTEHGTTATIT***PQAPTTEIQLT*** ^**176**^ EP8/E	^**165**^ ***PQAPTTEIQLT***DYG***ALTL*** ^182^ EP9/E	NP
^**180**^ ***ALTL***DCSPRTGLD^192^ EP10/E		

^218^LPWTS***GASTSQETW***NR^233^ EP11/E	^**223**^ ***GASTSQETW*** ^**231**^ EP12/E	NP

^**242**^ ***TAHAKKQ*** ^**248**^ EP14/E	^**237**^LVTFK***TAHAKKQ***EVV***VLGS*** ^255^ EP13/E	NP
^**252**^ ***VLGS***QEGAMHTALTGATEIQTSGTTTI^278^ EP15/E		

^**306**^ ***FKLEKEVAETQHGT*** ^319^ EP18/E	^***287***^ ***LKMDKLTLKGMS***YVMCTGS***FKLEKEVA*** ^**313**^ EP17/E	^279^FAGHLKCR***LKMDKLTLKGMS*** ^298^ EP16/E
^**326**^ ***YEGTDAPCKIPFSTQDEKGVTQNG***RLIT^353^ EP21/E	^322^ ***VQIKYEGTDAPCKIPFSTQDEKGVTQNG*** ^349^ EP20/E	^310^ ***KEVAETQHGT***VL***VQIKYEGT*** ^**329**^ EP19/E

^356^ ***PIVTDKEKPV***NIEAE***PPFGES*** ^376^ EP22/E	^356^ ***PIVTDKEKPV*** ^365^ EP23/E	NP
^**380**^ ***IGVGEKA*** ^**386**^ EP25/E	^**371**^ ***PPFGES***YIV***IGVGEKA***LKLSWF***KKGSSIGKMF*** ^**402**^ EP24/E	
^**393**^ ***KKGSSIGKMF***EATARGA^409^ EP26/E		

^**417**^ ***DTAWDFGSIGGVFT*** ^**430**^ EP28/E	^**425**^ ***IGGVFTSVGKL***VHQIFGTAYG^445^ EP29/E	^**416**^G***DTAWDFGSIGGVFTSVGKL*** ^**435**^ EP27/E

^**469**^ ***NSRSTSL*** ^475^ EP32/E	^***454***^TMKIG***IGVLLTWLGLNSRSTSLSM***SCIAVGIITLY^488^ EP30/E	^**459**^ ***IGVLLTWLGLNSRSTSLSM*** ^**478**^ EP31/E

NP: no prediction in the relevant region; bold italics indicates sequences that overlap between the predicted epitopes by three different tools.

**Table 4:** Overlapping epitopes of the prM protein.

BepiPred	Ellipro	SVMTriP
^**4**^ ***TTRGGE***PHMIV***SKQERG*** ^**20**^ EP2/prM	^**1**^FHL***TTRGGE*** ^**9**^ EP1/prM	NP
	^**15**^ ***SKQERG***KS^**22**^ EP3/prM	

^**44**^LCEDTMTYKCP***RITEAEPDDVD***CWCNATDTWVTYGTCSQTGEHRRDKRSV^**93**^ EP6/prM	^**55**^ ***RITEAEPDDVD*** ^**65**^ EP7/prM	NP

^**125**^ ***TWALR*** ^**129**^ EP10/prM ^**145**^ ***GTSITQ*** ^**150**^ EP13/prM	^**115**^GAWKQIQRVE***TWALRHPGFTVILALFLAHAIGTSITQ***KGIIFILLMLVTPS^**165**^ EP12/prM	^**127**^ ***ALRHPGFTILALFLAHAIGT*** ^**146**^ EP11/prM

NP: no prediction in the relevant region; bold italics indicates sequences that overlap between the predicted epitopes by three different tools.

**Table 5:** WebLogo of predicted epitopes on E protein.

Epitope ID	WebLogo result^*∗*^
EP1/E	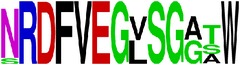

EP2/E	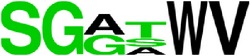

EP3/E	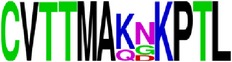

EP4/E	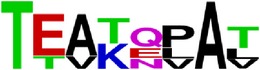

EP5/E	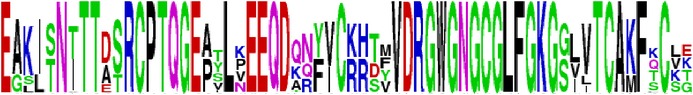

EP6/E	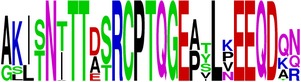

EP7/E	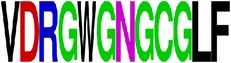

EP8/E	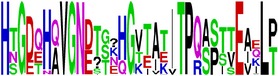

EP9/E	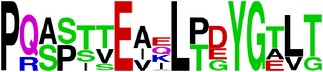

EP10/E	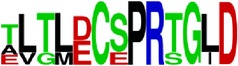

EP11/E	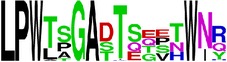

EP12/E	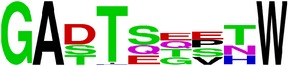

EP13/E	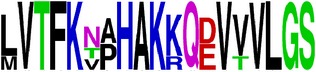

EP14/E	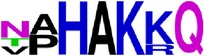

EP15/E	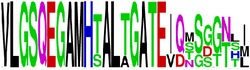

EP16/E	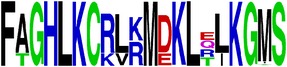

EP17/E	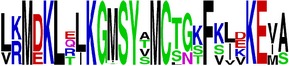

EP18/E	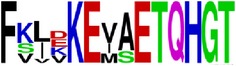

EP19/E	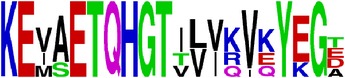

EP20/E	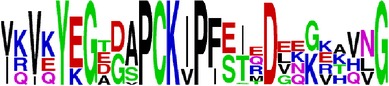

EP21/E	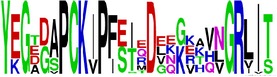

EP22/E	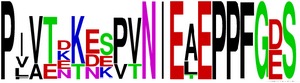

EP23/E	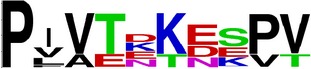

EP24/E	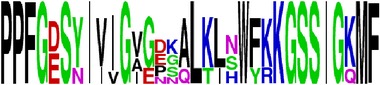

EP25/E	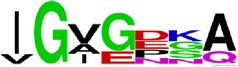

EP26/E	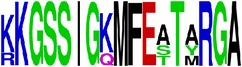

EP27/E	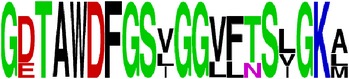

EP28/E	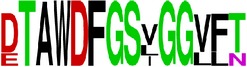

EP29/E	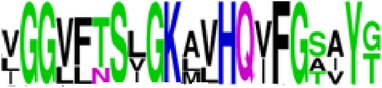

EP30/E	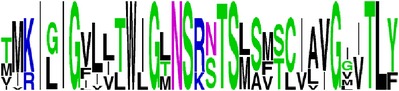

EP31/E	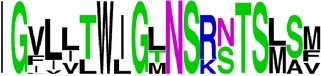

EP32/E	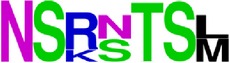

^*∗*^The logo consists of stacks of letters, one stack for each position in the sequence. The overall height of each stack indicates the sequence conservation at that position (measured in bits), whereas the height of symbols within the stack reflects the relative frequency of the corresponding amino acid at that position. Amino acids have colors according to their chemical properties; polar amino acids (G, S, T, Y, C, Q, and N) are shown as green, basic amino acids (K, R, and H) as blue, acidic amino acids (D and E) as red, and hydrophobic amino acids (A,V, L, I, P, W, F, and M) as black.

**Table 6:** WebLogo of predicted epitopes of prM protein.

Epitope ID	WebLogo results^*∗*^
EP1/prM	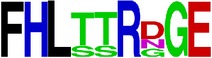

EP2/prM	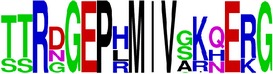

EP3/prM	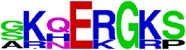

EP4/prM	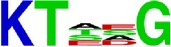

EP5/prM	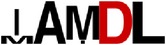

EP6/prM	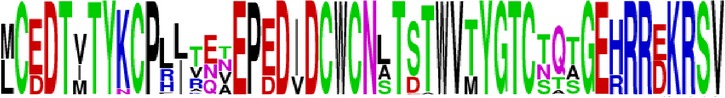

EP7/prM	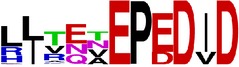

EP8/prM	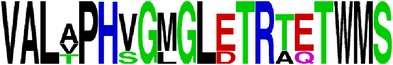

EP9/prM	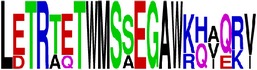

EP10/prM	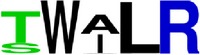

EP11/prM	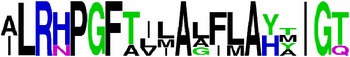

EP12/prM	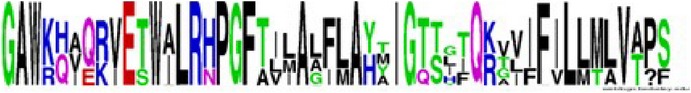

EP13/prM	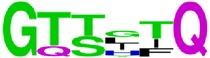

^*∗*^The logo consists of stacks of letters, one stack for each position in the sequence. The overall height of each stack indicates the sequence conservation at that position (measured in bits), whereas the height of symbols within the stack reflects the relative frequency of the corresponding amino acid at that position. Amino acids have colors according to their chemical properties; polar amino acids (G, S, T, Y, C, Q, and N) are shown as green, basic amino acids (K, R, and H) as blue, acidic amino acids (D and E) as red, and hydrophobic amino acids (A,V, L, I, P, W, F, and M) as black.
